# Advanced‐Stage Gonadal Dysgerminoma in a Patient With a Previous Diagnosis of Familial Swyer Syndrome: A Very Rare Genetic Entity

**DOI:** 10.1155/carm/3312911

**Published:** 2026-01-17

**Authors:** Süleyman Cemil Oğlak, Nilgün Söğütçü, Sedat Akgöl, Özgür Adıgüzel, Hümeyra Elif Ateş Eminoğlu, Gizem Güzel, Veysi Balcı, Emine Acar, Abdullah Acar, Fuat Bozan

**Affiliations:** ^1^ Department of Obstetrics and Gynecology, Gazi Yaşargil Training and Research Hospital, Health Sciences University, Diyarbakır, Turkey, sbu.edu.tr; ^2^ Department of Pathology, Gazi Yaşargil Training and Research Hospital, Health Sciences University, Diyarbakır, Turkey, sbu.edu.tr; ^3^ Department of Gynecologic Oncology, Gazi Yaşargil Training and Research Hospital, Health Sciences University, Diyarbakır, Turkey, sbu.edu.tr

**Keywords:** dysgerminoma, familial swyer syndrome, gonadal dysgenesis, surgical debulking

## Abstract

**Introduction:**

Swyer syndrome is a genetic abnormality characterized by a 46,XY karyotype in a phenotypically female individual. Affected individuals typically have average or tall stature, unambiguous genitalia at birth, the presence of Müllerian structures, and bilateral streak gonads. Familial Swyer syndrome is extremely rare, and we identified only two case reports describing families with two and three sisters affected by this syndrome. Individuals with Swyer syndrome have an increased risk of developing gonadal malignancies.

**Case:**

A 19‐year‐old patient with primary amenorrhea, who had been followed up with a diagnosis of Swyer syndrome, was referred to our hospital due to a complaint of progressive abdominal distension and a 5‐month history of lower abdominal pain. Karyotype analysis had been performed at another hospital when her older sibling was diagnosed with Swyer syndrome. However, she did not undergo bilateral gonadectomy after the diagnosis was confirmed, as her family postponed the procedure due to the COVID‐19 pandemic. Surgical debulking of ovarian cancer was performed, and histopathology revealed a dysgerminoma, FIGO Stage IIIC.

**Conclusion:**

Early diagnosis of Swyer syndrome is essential, considering the significant risk of malignant gonadal tumors that may arise at an early age. Prepubertal female siblings of patients diagnosed with Swyer syndrome should be screened. Early diagnosis and prompt prophylactic gonadectomy can allow for a conservative treatment plan that may preserve fertility and improve patient survival.

## 1. Introduction

Swyer syndrome was first described in 1955 by G.I.M. Swyer, who reported two phenotypically female cases with a male karyotype, female external genitalia, tall stature, and primary amenorrhea. These patients had normal growth of axillary and pubic hair and a normal vagina and cervix, although the breasts were underdeveloped, accompanied by a small uterus and nonpalpable adnexa [1]. This disorder was initially termed male pseudohermaphroditism and was later classified among congenital disorders of sex development (DSDs), which are characterized by disrupted anatomical, gonadal, or chromosomal sex development [2].

Complete or pure gonadal dysgenesis, known as Swyer syndrome, is a genetic abnormality characterized by a 46,XY karyotype in a phenotypically female individual. It is a rare disorder that occurs in approximately 1 in 80–100,000 live births [3, 4]. Affected individuals typically have average or tall stature, unambiguous genitalia at birth, the presence of Müllerian structures, and bilateral streak gonads instead of ovaries or testes due to gonadal dysgenesis [5]. Normally, the sex‐determining region Y (*SRY*) gene on the Y chromosome initiates the gene expression cascade that transforms the undifferentiated gonad into a testis. Anti‐Müllerian hormone (AMH), released by testicular Sertoli cells, triggers the regression of the Müllerian ducts in the fetus [6]. Approximately 15% of Swyer syndrome cases result from *SRY* point mutations, another 15% from *SRY* deletions caused by aberrant X/Y recombination, while nearly 70% of all cases have unidentified causes, which may be due to mutations in other testis‐determining genes [7].

The vast majority of Swyer syndrome cases first seek medical care for primary amenorrhea and other signs of delayed puberty, such as the absence of secondary sexual characteristics, due to the lack of gonadal hormone production. These individuals typically present with underdeveloped breasts but normal pubic and axillary hair. The external genitalia are phenotypically female, the vagina is normal or small, and the uterus is small or rudimentary [2]. Exogenous hormonal replacement therapy (HRT) is used to induce puberty and maintain female secondary sexual characteristics [8].

Swyer syndrome may also manifest in later adulthood with gonadal tumors. Swyer syndrome cases have an increased risk of developing gonadal malignancies. The neoplastic transformation risk of germ cells in dysgenetic gonads is common with an estimated risk of 20–30%. The most common tumor in these cases is bilateral gonadoblastoma [5, 8]. Although gonadoblastoma is considered a benign ovarian neoplasm, its association with malignant germ cell tumors (GCTs), especially dysgerminoma, has been observed in up to 60% of cases. Therefore, routine prophylactic bilateral gonadectomy is strongly recommended for Swyer syndrome cases, typically during adolescence [9, 10].

Familial Swyer syndrome is a rare occurrence in the literature. Ates et al. reported two sisters with Swyer syndrome, one of whom remained amenorrheic despite HRT [11]. Banoth et al. described three sisters from the same family who had Swyer syndrome with three different genotypes; the oldest sibling died from advanced‐stage ovarian malignancy, the second sister (the index patient) had dysgerminoma, and the third sister also had primary amenorrhea [12]. Herein, we present the case of a 19‐year‐old woman with Swyer syndrome, who also had a sister with the same diagnosis, and whose family postponed gonadectomy during the COVID‐19 pandemic. She presented to our hospital with an adnexal mass that was diagnosed as gonadal dysgerminoma.

## 2. Case Report

A 19‐year‐old patient with primary amenorrhea, who had been followed up with a diagnosis of Swyer syndrome, was referred to our hospital due to complaints of progressive abdominal distension and a 5‐month history of lower abdominal pain. Ultrasound (US) examination before referral revealed a complex abdominopelvic mass thought to originate from the gonad. On physical examination, the abdomen was nontender, and a fixed abdominopelvic mass was palpable. US examination documented a solid mass lesion measuring 25 × 20 cm, extending from the pelvis to the upper level of the umbilicus. She had no history of systemic disease or previous surgery. She was not taking any medication and did not smoke.

Her family history revealed an older sibling diagnosed with Swyer syndrome. This older sister presented to a private clinic with primary amenorrhea, underdeveloped breasts, and scant axillary and pubic hair at 15 years of age. Laboratory examination of the sister showed decreased serum estradiol levels and increased serum FSH and LH levels. US evaluation revealed a hypoplastic uterus. Further investigation with a karyogram demonstrated a 46,XY karyotype. This diagnosis was followed by bilateral gonadectomy at another hospital. She has been receiving estrogen and progesterone replacement therapy since gonadectomy and currently experiences regular monthly menstrual bleeding. Also, the development of secondary sexual characteristics was achieved through HRT.

**Figure 1:**
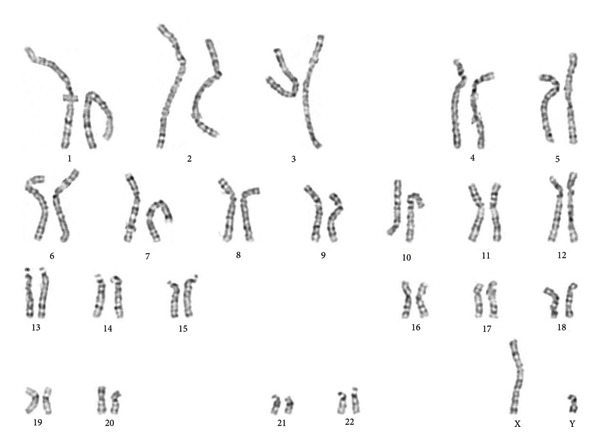
Karyotype analysis of the patient shows a 46,XY chromosomal pattern, consistent with complete gonadal dysgenesis.

Given the older sister’s history of Swyer Syndrome, clinicians advised the family to have her younger sister (our patient) evaluated. Further evaluation at the age of puberty (14 years old) revealed a 46,XY karyotype (Figure 1). Clinicians recommended prophylactic gonadectomy due to the risk of gonadal malignancy, but her family postponed the surgery because of the COVID‐19 pandemic. She also did not receive HRT following diagnosis. At the admission time to our hospital, she had primary amenorrhea, female external genitalia, a normal amount of axillary and pubic hair, tall stature, and a normal vagina and cervix; however, the breasts were underdeveloped, and the uterus was rudimentary.

**Figure 2:**
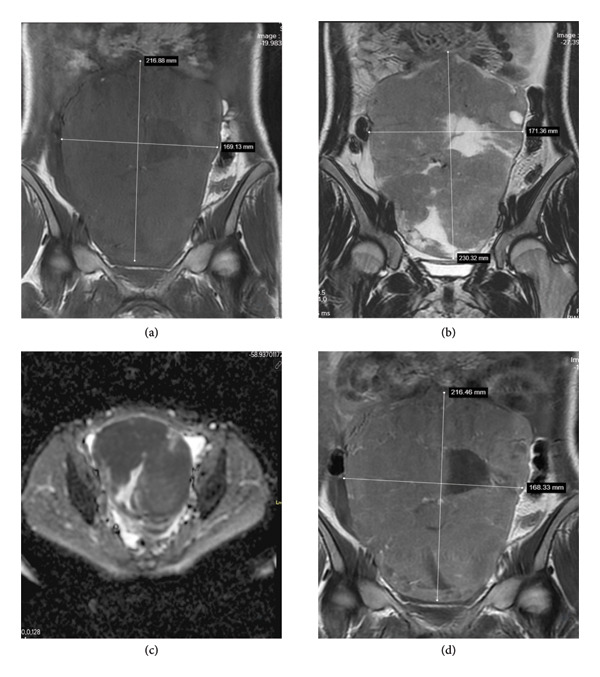
Magnetic resonance imaging (MRI) findings of the pelvic mass. (a) Coronal noncontrast T1‐weighted image demonstrates a large, heterogeneous, predominantly hypointense mass extending from the pelvic cavity to the level of the umbilicus, measuring approximately 21 × 19 cm. (b) Coronal T2‐weighted image reveals a heterogeneous mass with mixed cystic and solid components, showing variable signal intensity. (c) Diffusion‐weighted imaging (DWI) and corresponding apparent diffusion coefficient (ADC) map show restricted diffusion within the solid components of the mass, suggestive of high cellularity. (d) Postcontrast coronal T1‐weighted image demonstrates marked enhancement of the solid components.

Laboratory examination of our patient revealed normal serum levels of CEA, AFP, and CA 19–9 but elevated levels of β‐human chorionic gonadotropin (β‐HCG) (492.4 IU/mL, reference range between 0 and 5 IU/mL), CA 15–3 (50.9 IU/mL, reference range between 0 and 25 IU/mL), and CA 125 (985.7 IU/mL, reference range between 0 and 35 IU/mL). Magnetic resonance imaging (MRI) demonstrated a complex mass with cystic and solid areas, likely originating from the right streak gonad, measuring 230 × 171 × 85 mm, and extensive ascites in the abdomen (Figure 2). Positron emission tomography/computed tomography (PET/CT) showed increased ^18^F‐FDG uptake in the pelvic mass, enlarged abdominal lymph nodes, omentum, malignant ascites, and bilateral pleural effusion. The mass was evaluated as a solid pelvic tumor, and surgery was recommended for further assessment.

**Figure 3:**
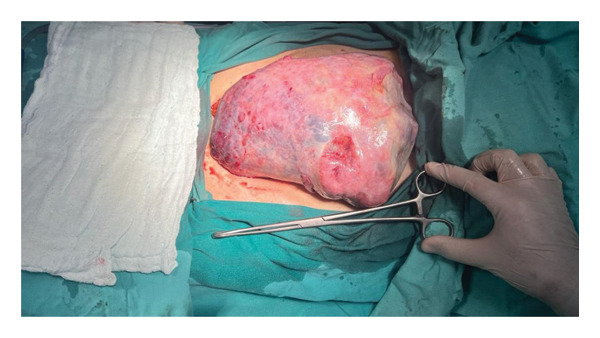
Intraoperative image showing a size of 24 × 19 cm tumor arising from the streak gonad.

An exploratory laparotomy was performed. Intraoperative findings revealed an approximately 24‐cm malignant mass in the right streak gonad (Figure 3). Other surgical findings were as follows: The contralateral streak gonad was grossly normal. The uterus was rudimentary, measuring approximately 6 × 3 × 1 cm. The bilateral fallopian tubes were slender and free of adhesions. Approximately 3000 mL of peritoneal free fluid (ascites) was present in the pelvis and abdomen. The paraaortic lymph nodes were enlarged with soft consistency, forming packages approximately 6 × 4 cm in diameter. The surfaces of the other abdominal organs were smooth, without evidence of malignancy, and no nodules were observed on the omentum.

Total abdominal hysterectomy, bilateral gonadectomy, bilateral salpingectomy, infracolic omentectomy, and paraaortic lymphadenectomy were performed. Additionally, 50 mL of free fluid was collected for pathological evaluation. Pathology revealed dysgerminoma in the right gonad. The peritoneal fluid sample analyzed was positive for malignancy. Histologic analysis of the left gonad showed an infantile gonad without evidence of malignancy.

According to the FIGO staging system, the tumor was classified as Stage IIIC, and adjuvant chemotherapy consisting of four cycles of bleomycin, etoposide, and platinum (BEP) was administered.

### 2.1. Pathology

It is an encapsulated ovarian resection specimen, weighing 1925 g and measuring 24 × 19 × 5 cm. Capsule integrity was found to be impaired. The cut surface was tan‐white in color, had a solid lobulated appearance, and showed areas of hemorrhage and necrosis in places. The uterus weighed 32 g and measured 6 × 3 × 0.9 cm. No pathological features were observed in the bilateral fallopian tubes. The other gonad appeared as a streak gonad.

We fixed samples from the tumor and other tissues in 10% formalin and embedded them in paraffin. Four micrometer‐thick sections were taken from each paraffin block, and one section was stained with hematoxylin and eosin (H&E). Immunohistochemical (IHC) staining was performed on some of the tissues obtained from the tumor. IHC stains were processed with the Ventana ultraView Universal DAB Detection Kit (REF 760–500, Ventana Medical Systems, Inc., Tucson, AZ, USA) using the Ventana BenchMark XT automated IHC/ISH staining system.

**Figure 4:**
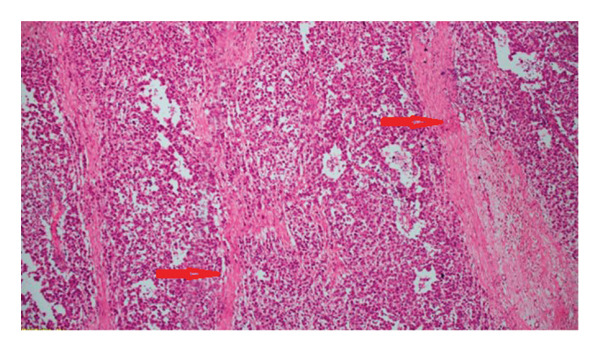
Uniform round‐appearing tumor cells separated by fibrous bands (shown with red arrows, H&E × 100).

**Figure 5:**
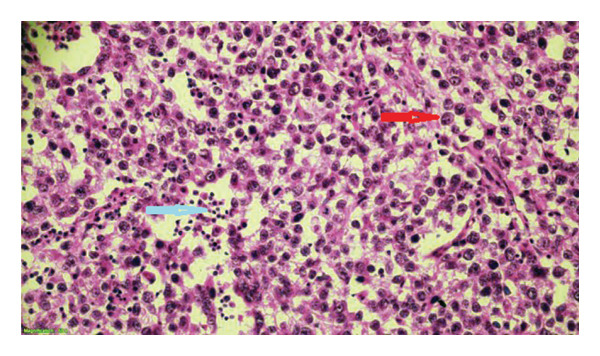
Lymphoid cells (shown with a blue arrow) among uniform polygonal tumor cells with prominent nucleolus, oval round nucleus, large clear or eosinophilic cytoplasm, and well‐defined cell borders (shown with a red arrow, H&E ×400).

**Figure 6:**
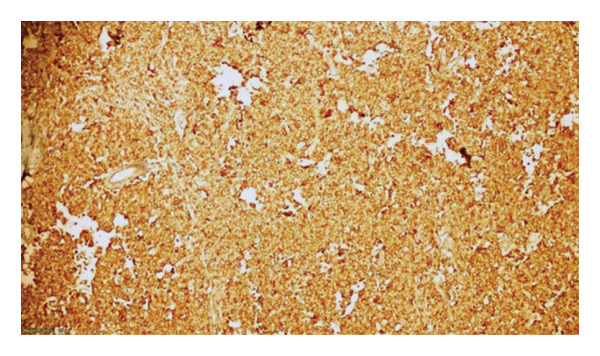
Immunohistochemically positive membranous and cytoplasmic staining with PLAP (× 100).

**Figure 7:**
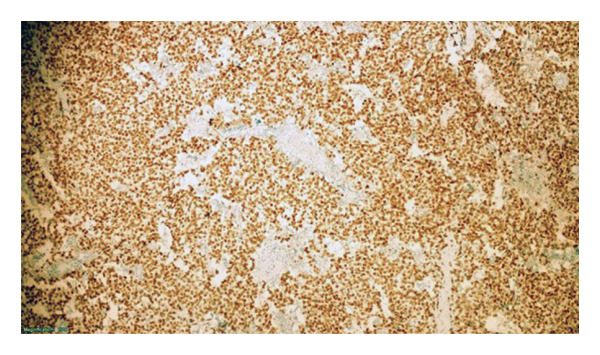
Immunohistochemically positive nuclear staining with SALL4 (× 100).

Microscopic examination revealed that the tumor consisted of atypical cells separated by fibrous septa containing lymphocytes, histiocytes, and plasma cells. The atypical cells were uniform polygonal cells with oval‐to‐round nuclei containing at least one nucleolus, large clear or eosinophilic cytoplasm, and well‐defined cell borders, forming irregular clusters and trabeculae (Figures 4 and 5). The stroma was loose and delicate, with several areas of necrosis accompanied by dystrophic calcification in focal regions. Tumor cells showed membranous and cytoplasmic staining with D2‐40 and PLAP (Figure 6), nuclear staining with SALL4 (Figure 7), and membranous staining with CD117. Tumor cells did not show staining for EMA, CD30, PanCK, AFP, or GATA3. The Ki‐67 proliferation index was high (about 80%).

Since the B‐HCG level was moderately high in the patient, numerous samples were taken from the tumor to search for possible syncytiotrophoblastic cells, and suspicious areas were stained with B‐HCG immunohistochemically. However, no syncytiotrophoblastic cells were found.

No pathological features were observed in the cervix and endometrium.

Tumor invasion was observed in three of 4 lymph nodes, the largest measuring 6.5 × 4.5 × 2.5 cm. Omental tumor involvement was not observed. Atypical cells consistent with dysgerminoma were identified in the smears obtained from the ascitic fluid in the abdomen.

## 3. Discussion

Individuals with Swyer syndrome are phenotypic females with hypoplastic gonads that lack germ cells or ovarian follicles. These patients are usually raised as females. They frequently present and are diagnosed with primary amenorrhea and delayed pubertal development in early adolescence because of the absence of hormonal activity of the gonads. The etiology of this syndrome is considered to be a deletion on the short arm of the Y chromosome involving the *SRY* gene, a mutation affecting *SRY* function, and most commonly, mutations in various genes that result in the inhibition of *SRY* function [3, 13]. Accordingly, the Wolffian ducts of these cases undergo atrophy, and patients do not form testes but demonstrate female external genitalia. Also, Müllerian ducts form the fallopian tubes, uterus, cervix, and proximal third of the vagina because of the lack of testosterone and AMH [10]. As expected, our case and her older sibling demonstrated female external genitalia, a uterus, and underdeveloped breasts.

Swyer syndrome cases show normal female levels of androgen, low levels of estrogen, and increased gonadotropin levels secondary to gonadal failure, which are indicative of hypergonadotropic hypogonadism. The gonads exhibit fibrous tissue that resembles ovarian stroma without follicles. The uterus is rudimentary because of the low estrogen levels. Early diagnosis is essential for these cases, considering the risk of gonadal malignancy, the need for early administration of HRT for puberty induction, and the improvement of bone mineral density. HRT provides menstrual bleeding episodes and supports female secondary sex characteristics, such as body hair and breast development. Therefore, HRT should be administered to these cases as soon as possible following the diagnosis [5]. Also, the literature reports a small number of Swyer syndrome cases with a hypoplastic uterus, in which pregnancies have been achieved through oocyte donation. The outcomes of these pregnancies are similar to those of 46,XX individuals with ovarian failure [14, 15].

In the differential diagnoses, women with primary amenorrhea should be evaluated for several possibilities. Normally developed ovaries with hormonal function and a 46,XX karyotype distinguish Mayer–Rokitansky–Küster–Hauser syndrome from Swyer syndrome. Complete androgen insensitivity syndrome is also considered. The karyotype of this syndrome is XY, as in Swyer syndrome; however, radiologic imaging displays the presence of testes and the absence of a uterus in these cases. Thus, karyotyping should be performed in any patient with pubertal delay and increased gonadotropins. Differentiating Swyer syndrome from other causes of primary amenorrhea allows for early initiation of HRT with its benefits and provides the possibility of pregnancy through ART with oocyte donation [14, 16].

Patients with Swyer syndrome have a significantly increased risk of developing gonadoblastoma and associated malignant GCTs [9]. This syndrome carries the highest risk of GCTs among cases with Y‐chromosome–related DSD, reaching up to 23.3% [17]. Malignant transformation in the gonadal ridge might occur even at younger ages, and this risk increases with age, ranging from 5% at 15 years, 27.5% at 30 years, and up to 50% after 40 years [8]. Overall, the cumulative lifetime risk of developing a gonadal tumor, especially gonadoblastoma, is estimated at 30% [12]. More than 40% of gonadoblastomas are bilateral and 96% arise in the dysgenetic gonads of individuals with a Y chromosome. Although gonadoblastomas are benign tumors, overgrowth of the germinal component leading to malignant transformation, most commonly dysgerminoma, occurs in 50–60% of cases [6]. Therefore, intraabdominal gonads should be surgically removed as soon as the diagnosis is confirmed because of the well‐established risk of gonadal malignancy.

Dysgerminoma is an aggressive neoplasm that rapidly grows, invades ovarian tissue, and infiltrates adjacent organs, with metastases to distant organs and frequent recurrences [8]. Dysgerminoma is the most common ovarian malignant GCT, comprising about 45% of malignant GCTs and nearly 1% of ovarian malignancies [18]. Dysgerminoma is commonly unilateral, but bilateral involvement is detected in about 10–15% of cases. It differs in this regard from other malignant ovarian GCTs, which are typically unilateral [3]. Dysgerminomas are commonly present in their pure form, while less than 15% of cases contain other malignant germ cell elements. About two‐thirds of women with dysgerminoma are diagnosed at Stage I [18]. Most patients present with large adnexal masses. However, postoperative diagnosis of dysgerminoma in a streak gonad has also been reported in the literature. Thus, prompt prophylactic gonadectomy is highly recommended for Swyer syndrome cases, regardless of gonadal volume [9].

Familial Swyer syndrome is extremely rare, and we identified only two case reports describing 2 and 3 sisters from the same families with this syndrome. Familial screening with a karyotype study should be offered to all prepubertal asymptomatic female relatives of affected cases, which is of significant importance for the abovementioned reasons. Karyotype analysis was performed on our patient at another hospital when her older sibling was diagnosed with Swyer syndrome [11, 12].

In dysgerminoma, ^18^F‐FDG PET/CT can be a valuable preoperative imaging tool because these tumors typically demonstrate high metabolic activity and FDG avidity. PET/CT may aid in evaluating the extent of disease, identifying metabolically active lymph nodes, and detecting distant metastatic sites that may not be fully appreciated on conventional imaging. This can help guide surgical planning, particularly in advanced‐stage disease. Studies of malignant ovarian GCTs, including dysgerminomas, have reported high sensitivity of FDG–PET/CT for detecting metabolically active tumor sites, supporting its use as an adjunct to CT and MRI in pretreatment evaluation [19]. In our case, PET/CT was similarly performed preoperatively to assess the metabolic activity of the ovarian mass and to evaluate potential metastatic involvement.

Because of the high risk of tumor development in the gonads of Swyer syndrome cases, early diagnosis is of great importance. Also, bilateral gonadal resection should be performed as early as possible, ideally before tumor development and metastasis [20]. Our case did not undergo bilateral gonadectomy after the diagnosis was confirmed because her family postponed the procedure due to the COVID‐19 pandemic. As is known, due to the financial difficulties brought by the pandemic, there has been a sharp decline in access to non–COVID‐19 healthcare. She was admitted due to an ovarian mass detected on US and MRI, with high serum β‐HCG and CA 125 levels, along with a confirmed diagnosis of Swyer syndrome, and underwent laparotomy for debulking surgery. Since dysgerminomas are sensitive to chemotherapy and the use of chemotherapy has been related to a significant improvement in patient survival in previous reports [2], a platinum‐based regimen was administered to our case.

## 4. Conclusion

Early diagnosis of Swyer syndrome is crucial, considering the high risk of malignant gonadal tumors that might arise at an early age. Prepubertal female siblings of patients diagnosed with Swyer syndrome should be screened. Early diagnosis and prompt prophylactic gonadectomy provide a conservative treatment plan that may preserve fertility and improve survival.

## Ethics Statement

The patient was treated in accordance with the Declaration of Helsinki. Written informed consent for publication was obtained from both the patient and her parents.

## Conflicts of Interest

The authors declare no conflicts of interest.

## Funding

No funding was received for this manuscript.

## Data Availability

The data that support the findings of this study are available upon request from the corresponding author. The data are not publicly available due to privacy or ethical restrictions.
